# Tolerability of lopinavir versus dolutegravir in children and adolescents with HIV

**DOI:** 10.1097/QAD.0000000000004432

**Published:** 2026-03-10

**Authors:** Jacob Blankenberger, Akash Devendra, Meenakshi Bakaya, Tristan Lee, Teresa L. F. Steffy, Thithili Makhesi, Charlie J. Gilbride, Jennifer M. Belus, Lineo Thahane, Nadine Tschumi, Nthuseng B. Marake, Tapiwa Tarumbiswa, Reto Huber, Frédérique Chammartin, Niklaus D. Labhardt, Jennifer A. Brown

**Affiliations:** a Division of Clinical Epidemiology, Department of Clinical Research, University Hospital Basel, Basel; b University of Basel, Basel, Switzerland; c Baylor College of Medicine Children's Foundation Lesotho, Maseru, Lesotho; d Department of Pediatrics; e Baylor International Pediatric AIDS Initiative at Texas Children's Hospital, Baylor College of Medicine, Houston, TX, USA; f Health Psychology Research Ltd., Egham, Surrey, Great Britain; g Ministry of Health, Maseru, Lesotho; h Child Development Center, University Children's Hospital Zurich; i Department of Child and Adolescent Psychiatry and Psychotherapy, Psychiatric Hospital Zurich, University of Zurich, Zurich, Switzerland.

**Keywords:** HIV, integrase inhibitors, patient-reported outcome measures, pediatrics, protease inhibitors, sleep

## Abstract

**Background:**

Children and adolescents with HIV previously taking ritonavir-boosted lopinavir (LPV/r)-based antiretroviral therapy (ART) were recently programmatically transitioned to dolutegravir (DTG)-based ART in Lesotho, southern Africa. We investigated associated changes in treatment satisfaction and potential side effects.

**Methods:**

This single-center prospective cohort study enrolled participants younger than 18 years transitioned from LPV/r-based to DTG-based ART during the national programmatic DTG rollout in 2022–2023. Virally suppressed participants who were at least 6 years old and able to handle a sleep diary and actigraphy were eligible for additional sleep monitoring. Enrollment occurred 2 weeks before (with actigraphy) or at (without actigraphy) transition with follow-up until 4 weeks posttransition. Co-primary endpoints were change in treatment satisfaction assessed with the HIV Treatment Satisfaction Questionnaire change version (HIVTSQc; Teen and Parent versions) at 4 weeks, and difference in mean sleep period length over a 2-week period before and after transition (only actigraphy participants). Secondary endpoints assessed treatment satisfaction status, gastrointestinal symptoms, depressive symptoms, and additional sleep measures.

**Results:**

Among 245 participants with transition and 4-week data, 115 (47%) were girls and median age was 11.1 (interquartile range 8.9–13.6) years. HIVTSQc outcomes favored DTG, with 88/92 (96%) HIVTSQc-Teen and 149/151 (99%) HIVTSQc-Parent responses indicating being ‘much more satisfied now’ posttransition. Among 69 (28%) actigraphy participants, mean sleep period length was 9.0 h [standard deviation (SD) 1.0] before and 9.2 h (SD 1.0) 2–4 weeks posttransition [mean difference 0.2, 95% confidence interval (CI) 0.0–0.4]. Secondary outcomes did not change meaningfully.

**Conclusion:**

Observed treatment satisfaction and tolerability support the rollout of DTG in pediatric HIV care.

## Introduction

Globally, an estimated 2.6 million children and adolescents (aged 0–19 years) are living with HIV. Compared with adults, children and adolescents with HIV receiving antiretroviral therapy (ART) have high rates of treatment failure [1]. Limited well tolerated, convenient pediatric ART regimens and adherence challenges have long contributed to poor treatment outcomes [2].

Since 2018, ART containing the integrase strand transfer inhibitor (INSTI) dolutegravir (DTG) has been recommended as the preferred regimen type for adolescents and for children in age and weight groups with approved DTG dosing [3]. DTG has been rolled out in several steps, phasing out previous WHO-recommended regimens including ART containing the protease inhibitor ritonavir-boosted lopinavir (LPV/r). LPV/r was previously recommended as first-line ART for children initiating treatment below 3 years of age, and as second-line ART upon treatment failure with a nonnucleoside reverse transcriptase inhibitor (NNRTI)-based regimen [3–5], and was briefly the most common ART regimen among children below 15 years of age in low-income and middle-income countries [6,7].

Although effective, LPV/r-based ART must be taken twice daily, can entail complex formulations (including combinations of pills with pellets, granules, and syrup), and is not available as a fixed-dose combination [2]. Gastrointestinal symptoms (primarily diarrhea) and metabolic disturbances have also been described [8,9].

In adults, DTG is available as a convenient once-daily fixed-dose combination and has demonstrated noninferior or superior virological efficacy compared to protease inhibitors [10], NNRTIs [11], and other INSTIs [12]. It also has a lower potential for drug interactions [13] and a high resistance barrier [10–12], though DTG has been linked to sleep disturbances and neuropsychiatric symptoms such as depression [14–19]. Initial evidence in children regarding efficacy and side effects has been promising [20–25]. However, data are still limited, and no study has directly compared LPV/r-based to DTG-based ART in a pediatric population.

In Lesotho, southern Africa, most children previously on LPV/r-based ART transitioned to DTG between 2022 and 2023. This prospective cohort study assessed changes in reported treatment satisfaction and actigraphy-measured sleep duration among children and adolescents who transitioned from LPV/r-based to DTG-based ART in a primary care HIV clinic in Lesotho. Furthermore, emphasis was placed on reported depressive and gastrointestinal symptoms and additional indicators of sleep disturbance, the most frequently reported adverse events linked to DTG-based and LPV/r-based ART [8,14].

## Methods

### Study design

Tolerability of Lopinavir Versus Dolutegravir in Children and Adolescents with HIV (LoDoCA) was a single-center prospective cohort study assessing changes in treatment satisfaction, predefined potentially ART-related symptoms, and actigraphy-based sleep parameters among children and adolescents with HIV changing from LPV/r-based to DTG-based ART in the context of the programmatic DTG rollout in Lesotho. Reporting follows the Strengthening the Reporting of Observational studies in Epidemiology (STROBE) guidelines for cohort studies [26].

### Setting

Lesotho has the second-highest adult HIV prevalence globally at 19.3%, with 14 000 children and adolescents younger than 19 years living with HIV [1]. Prior to the programmatic DTG rollout, ART containing the NNRTI efavirenz (EFV) was the recommended first-line treatment for adults, adolescents, and children initiating ART while at least 3 years old. LPV/r-based ART was used as the first-line regimen for children aged at least 2 weeks and younger than 3 years old at ART initiation and maintained as children aged out of this group. Furthermore, it was used as second-line ART upon failure of first-line NNRTI-based ART [27]. From 2019 to 2021, in generic-accessible lower income and middle-income countries, LPV/r was the most common anchor drug for children and adolescents younger than 15 years [6,7].

Lesotho national guidelines were amended in 2019 to recommend DTG-based ART for most people with HIV [28]. DTG was initially available as a single-pill regimen with lamivudine (3TC) and tenofovir disoproxil fumarate (TDF) for people weighing at least 35 kg, and as a multipill regimen with 3TC and abacavir (ABC; preferred) or 3TC and zidovudine (AZT) for children and adolescents weighing 20–35 kg. Since 2022, ABC/3TC/DTG has become available for children aged at least 4 weeks and weighing at least 3 kg, with transition to TDF/3TC/DTG foreseen once they reach 35 kg [29].

In addition to availability for people newly initiating ART, people established on ART were transitioned to DTG-based ART in several stages. The main transition from NNRTI-based to DTG-based ART in Lesotho occurred through 2020 and has been described previously for children and adolescents [30] and adults [31,32].

### Participants

We enrolled children and adolescents with HIV aged less than 18 years on LPV/r-based ART and eligible for DTG transition under Lesotho's national guidelines [27,29]. A subset of participants fulfilling further criteria were additionally offered to participate in actigraphy-based sleep assessments. These criteria were: aged at least 6 years (due to actigraph wristband size and decreasing daytime sleep with increasing age [33]); having taken LPV/r-based ART for at least 12 weeks; a latest viral load result less than 50 copies/mL (measured within the last 36 weeks while taking LPV/r-based ART); willingness to wear an actigraph every night for at least seven nights; being judged by the study team member conducting the screening as able to wear an actigraph and fill in the sleep diary; and stated ability to attend all study visits. Exclusion criteria were: not being foreseen to transition to DTG-based ART, being enrolled in another incompatible study, and for actigraphy, intention to transfer out of the study site within 6 weeks or unavailability of an actigraph.

The study was conducted in the Centre of Excellence of the Baylor College of Medicine Children's Foundation Lesotho in Maseru, Lesotho, providing care to around 1300 children and adolescents with HIV. Participants were enrolled from 11 July 2022 to 9 August 2023 and followed up for 9 weeks or less (≤3 weeks before transition only for participants with actigraphy, and ≤6 weeks after transition).

### Procedures

Participants were recruited through convenience sampling. Participants without actigraphy were enrolled at DTG transition and had a 4-week follow-up visit. Participants with actigraphy were enrolled 2 weeks (window: 11–21 days) before DTG transition and had further visits at transition, and 2 (window: 11–21 days) and 4 weeks (window: 22–42 days) posttransition. Between visits, they were instructed to wear a GENEActiv (Activinsights Ltd., Kimbolton, United Kingdom) actigraph at night from before sleep onset until after wake-up. GENEActiv wrist-worn actigraphs are designed for continuous monitoring of physical activity and sleep patterns. Visits were aligned with the expected storage capacity of actigraphs, with a predefined measurement frequency of 60 Hz. Additionally, participants or their caregivers were asked to maintain a paper-based sleep diary, recording participants’ time of going to bed, sleep onset, and wake-up.

**Fig. 1:**
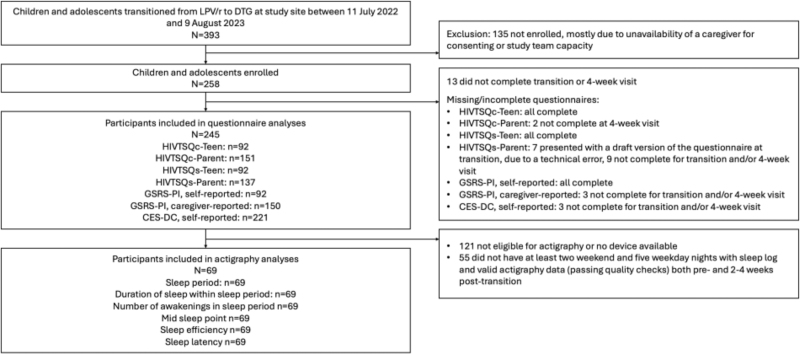
Flow chart.

DTG, dolutegravir; LPV/r, ritonavir-boosted lopinavir.

Clinical information was collected, and participant-reported or caregiver-reported standardized questionnaires completed at transition, 2 weeks posttransition (only for those with actigraphy), and 4 weeks posttransition. An overview of procedures is shown in Supplement 1 Figure 1.

### Outcomes

The co-primary endpoints were the self-reported/caregiver-reported change in treatment satisfaction [assessed with the HIV Treatment Satisfaction Questionnaire change version (HIVTSQc) for parents and teens (HIVTSQc-Parent/-Teen)] at 4 weeks posttransition, and difference in estimated length of the sleep period (measured by actigraphy) between pretransition and 2–4 weeks posttransition.

Secondary endpoints included in this analysis were: difference in self-reported or caregiver-reported treatment satisfaction status [HIVTSQ-Teen/HIVTSQ-Parent status versions (HIVTSQs-Teen/-Parent)], gastrointestinal symptoms (Gastrointestinal Symptom Rating Scale modified to the characteristics of protease inhibitors; GSRS-PI), and depressive symptoms (Center for Epidemiological Studies Depression Scale for Children; CES-DC) between transition and 4 weeks posttransition; and for those with actigraphy, difference in estimated duration of sleep within the sleep period, sleep fragmentation, latency, efficiency and midpoint between pretransition and 2–4 weeks posttransition.

### Measures

#### Questionnaires

The original HIVTSQs was designed to assess treatment satisfaction among adults receiving ART, rating items from ‘very dissatisfied’ to ‘very satisfied’ [34,35]. The HIVTSQc was designed for use after a regimen change, rating items from ‘much less satisfied now’ to ‘much more satisfied now’, and can help overcome ceiling effects in status measures of satisfaction [34,35]. For this study, we interviewed participants aged at least 12 years (HIV disclosure status for participants aged at least 12 years was confirmed at study enrollment) using the HIVTSQs/c-Teen, and caregivers of younger participants using the HIVTSQs/c-Parent.

Gastrointestinal symptoms such as pain and discomfort, nausea, reflux, flatus, and bowel movements including diarrhea were assessed using the GSRS-PI [36,37]. Wording was modified to create a caregiver-reported version for participants younger than 12 years, whereas the self-reported version was completed by participants aged at least 12 years (each rating 13 items on a 7-point Likert scale). The possible range for the GSRS-PI total score was 13–91, with higher scores indicating more severe symptoms.

Depressive symptoms were assessed using the CES-DC [38] through self-reporting by participants aged at least 6 years. The possible range for the CES-DC was 0–60, with higher scores indicating more severe symptoms. A threshold of 15 is often used to identify individuals who may be experiencing clinical depression [38].

Translation of the questionnaires followed a standardized process, which for the HIVTSQs/c-Parent and HIVTSQs/c-Teen was supported by Health Psychology Research Ltd. The linguistic validation process is described in Supplement 1.

#### Actigraphy

The length of the sleep period was defined as the estimated time from initial sleep onset to final wake-up. The duration of sleep within the sleep period subtracts estimated periods of wakefulness from the length of the sleep period. Sleep fragmentation was defined as the number of periods interpreted as wakefulness during the sleep window. The midpoint of sleep is the clock time halfway between sleep onset and is a marker of a person's chronotype. It is associated with quality of sleep and various other health indicators in children and adolescents [39,40]. Sleep efficiency measures the percentage of time spent sleeping relative to the total time in bed, while sleep latency is the duration between going to bed and sleep onset.

Sleep parameters were obtained from actigraphy raw data using GGIR (version 3.0-6) [41] in R statistical software (version 4.3.2, R Foundation for Statistical Computing, Vienna, Austria) [42], a package that includes a validated algorithm for sleep detection. Default settings were adapted to consider nights with more than 3 (rather than the default 16) h of valid data, as participants were only instructed to wear the devices at night. Additional data were collected using sleep logs by participants or their caregivers. Documented sleep onset and wake-up times were used as guiders to help the algorithm identify sleep periods [43], and participants’ bedtimes were used to calculate sleep efficiency and sleep latency. Supplement 1 Figure 2 shows a visualization of the GGIR-interpreted actigraphy data.

### Data management and statistical analysis

Routine clinical data and questionnaires were collected with Open Data Kit (ODK, Get ODK Inc., San Diego, USA) by trained research assistants. The sample size was not fixed, though we prespecified a target of at least 200 participants including at least 50 with actigraphy data.

For questionnaire-based outcomes, we included participants with data available at transition and at 4 weeks posttransition in analyses. For actigraphy analyses, we included participants with valid actigraphy data for at least 5 weekday nights and at least 2 weekend nights both pretransition and at 2–4 weeks posttransition. All actigraphy outcomes are reported as weighted averages of the mean values obtained for valid weekday and weekend nights, respectively.

Participant characteristics are described as median and interquartile range (IQR) and frequency and percentage, as appropriate. HIVTSQc-Teen and HIVTSQc-Parent outcomes are reported descriptively. For the HIVTSQs-Teen and -Parent, psychometric analysis was performed [44,45] to identify valid indicators of treatment satisfaction for both adolescents and caregivers, and to measure changes in treatment satisfaction as a latent variable (Supplement 2) [44]. Following psychometric assessment, four HIVTSQs-Parent items (Satisfied, Working well, Own life, Continue; Supplement 2 Table 1) and four HIVTSQs-Teen items (Satisfied, Easy-difficult, Fits your life, Continue; Supplement 2 Table 2) were identified as indicators of treatment satisfaction and included in the respective latent variable models. We selected transition as the reference time point (for model identification purposes), where the latent variable mean and variance are fixed at 0 and 1, respectively. The value at 4 weeks then inherently indicates the difference in treatment satisfaction levels between transition and 4 weeks. Two further items (Side effects and Pain/Discomfort) that could not be included in the models due to their lack of variance are reported descriptively as they were considered relevant as standalone items. For all other endpoints, we report the median and IQR or mean and standard deviation (SD) at transition and at 4-week posttransition, and the mean and 95% confidence interval (CI) of the difference. Statistical analysis was performed using R statistical software.

### Sensitivity analysis

We reanalyzed pretransition and 2–4 weeks posttransition actigraphy data following the approach outlined by Aili *et al.* [46] looking only at nights from Monday through Thursday (4 weekday nights) for participants with valid data for ≥4 of these nights at both timepoints. Additionally, actigraphy data from before transition were compared with the data 0–2 weeks (instead of 2–4 weeks) posttransition.

### Ethical considerations

This study was approved by the National Health Research Ethics Committee of Lesotho (ID37-2022) and the Institutional Review Board for Human Subject Research for Baylor College of Medicine and Affiliated Hospitals (H-51472). It was registered with ClinicalTrials.gov on 11 June 2022 (NCT05426421). After receiving study information in the local language, Sesotho, written informed consent was provided by study participants aged at least 16 years, or by caregivers of participants younger than 16 years. In addition, participants aged at least 6 and less than 16 years provided written informed assent. Informed consent and assent forms included the option to additionally consent or assent to sleep measurement by actigraphy. Participants or caregivers who were illiterate provided consent or assent, as appropriate, by thumbprint with witness signature. Among participants eligible and consenting to contribute actigraphy data, wearing actigraphs was only required at night to avoid unintended disclosure of HIV status. Transport costs were remunerated for study visits. Participants contributing actigraphy data or their caregivers were remunerated with LSL 150 (approximately USD 8) for each 2-week actigraphy period (i.e. maximally three times) during which they contributed actigraphy data.

## Results

### Participant characteristics

Between 11 July 2022 and 9 August 2023, 393 children and adolescents transitioned from LPV/r-based to DTG-based ART at the study site. Among these, 135 were not enrolled, mostly due to unavailability of a caregiver for consenting or study team capacity. Among 258 enrolled participants, 245 completed the transition and the 4-week visit and were included in the analysis. Of these 245 participants, 69 (28%) were eligible for and had complete data (sufficient sleep log and actigraphy data) to be included in the actigraphy analysis (Fig. 1). Participants’ clinical and sociodemographic characteristics are shown in Table 1 stratified by their inclusion in the actigraphy analysis and in Supplement 1 Table 1 stratified by age group. Additional characteristics regarding sleep/actigraphy measurements are shown in Supplement 1 Table 2. Overall, 115 (47%) participants were female individuals, median age was 11.1 years (IQR: 8.9–13.6) and median time on ART was 9.6 years (IQR: 7.2–12.4). Upon transition to DTG, the median number of daily pills decreased from 5 (IQR: 4–5) to 2 (IQR: 1–4).

**Table 1:** Participant characteristics at transition to DTG.

	Overall (*n* = 245)	Actigraphy (*n* = 69)
General characteristics		
Female sex [*n* (%)]	115 (47)	28 (41)
Age (years) (median [IQR])	11.1 [8.9–13.6]	11.7 [10.3–13.9]
Age category [*n* (%)]		
4 weeks to 6 years	21 (9)	0 (0)
6–11 years	132 (54)	41 (59)
≥12 years	92 (38)	28 (41)
Weight (kg) (median [IQR])^a^	26.6 [21.7–37.0]	30.1 [24.6–35.1]
Height (cm) (median [IQR])^a^	132 [119–146]	130 [114–145]
BMI category (WHO) [*n* (%)]^a^		
Severe malnutrition	7 (3)	4 (6)
Moderate malnutrition	21 (9)	9 (13)
Normal	196 (80)	49 (71)
Overweight	13 (5)	4 (6)
Obese	7 (3)	3 (4)
Clinical history		
Viral load measured at transition (copies/mL) [*n* (%)]^b^		
<50	200 (82)	62 (90)
50–1000	28 (12)	6 (9)
>1000	15 (6)	1 (1)
Documented last viral load result at screening (copies/mL) [*n* (%)]^c^		
<50	192 (78)	69 (100)
50–1000	20 (8)	0 (0)
>1000	10 (5)	0 (0)
Time since HIV diagnosis (years) (median [IQR])^d^	10.0 [7.5–12.7]	10.8 [8.9–12.5]
Time since ART initiation (years) (median [IQR])^e^	9.6 [7.2–12.4]	10.3 [8.5–11.8]
WHO stage at ART initiation [*n* (%)]^f^		
Stage 1	156 (70)	47 (76)
Stage 2	9 (4)	4 (6)
Stage 3	37 (17)	7 (11)
Stage 4	20 (9)	4 (6)
CD4^+^ cell count at ART initiation (cells/μl) (median [IQR])^g^	1061 [656–1830]	1072 [748–1953]
CD4^+^ percentage at ART initiation (cells/μl) (median [IQR])^h^	20 [13–29]	22 [13–32]
Immunodeficiency classification at ART initiation [*n* (%)]^i^		
None or not significant	32 (18)	14 (27)
Mild	17 (10)	7 (13)
Advanced	20 (11)	5 (10)
Severe	108 (61)	26 (50)
ART before transition		
Time since current ART regimen start (years) (median [IQR])^j^	7.6 [4.3–9.2]	7.9 [4.9–10]
ART regimen line before transition [*n* (%)]^a^		
First-line	193 (79)	53 (77)
Second-line	51 (30)	16 (23)
ART regimen before transition [*n* (%)]^a^		
ABC/3TC/LPV/r	169 (69)	38 (55)
AZT/3TC/LPV/r	72 (30)	30 (43)
TDF/3TC/LPV/r	3 (1)	1 (1)
ART formulation before transition [*n* (%)]		
Both pills and granules	21 (9)	0 (0)
Only pills	224 (91)	69 (100)
Number of pills/granules per day before transition (median [IQR])	5.0 [4.5–5]	5.0 [4.5–5]
ART after transition		
New ART regimen line [*n* (%)]^a^		
First-line	189 (77)	53 (77)
Second-line	54 (22)	16 (23)
Third-line	1 (0)	0 (0)
New ART regimen [*n* (%)]^a^		
ABC/3TC/DTG	176 (72)	52 (75)
TDF/3TC/DTG	67 (27)	17 (25)
AZT/3TC/DTG	1 (0)	0 (0)
Number of pills per day after transition (median [IQR])	2.0 [1–4]	2.0 [2–4]
Sociodemographic characteristics		
Primary caregiver [*n* (%)]		
Parent(s)	153 (62)	47 (68)
Other	92 (38)	22 (32)
Living situation [*n* (%)]		
With primary adult caregiver(s)	222 (91)	68 (99)
With other adult caregiver(s)	11 (4)	1 (1)
With older sibling who is <18 years	1 (0)	0 (0)
Boarding school or orphanage	11 (4)	0 (0)
Number of household members (including participant) (median [IQR])	4 [3–5]	5 [3–6]
Goes to school (= yes) [*n* (%)]^k^	224 (93)	66 (96)
Household has regular income (= yes) [*n* (%)]	180 (73)	53 (77)
Average monthly household income (in USD) [*n* (%)]^l^		
<57	106 (43)	24 (35)
57–287	116 (47)	39 (57)
>287	23 (9)	6 (9)

3TC, lamivudine; ABC, abacavir; ART, antiretroviral therapy; AZT, zidovudine; BMI, body mass index; DTG, dolutegravir; IQR, interquartile range; LPV/r, ritonavir-boosted lopinavir; TDF, tenofovir disoproxil fumarate; USD, United States Dollars.

a Missing for 1 (thereof 0 in actigraphy group).

b Missing for 2 (thereof 0 in actigraphy group), for three participants viral load was taken before transition (≤14 days), for two participants, viral load was taken after transition (≤5 days).

c Missing for 23 (thereof 0 in actigraphy group), as documented in the electronic medical record, used to determine eligibility to participate in actigraphy assessments.

d Missing for 11 (thereof 2 in actigraphy group).

e Missing for 9 (thereof 2 in actigraphy group).

f Missing for 23 (thereof 7 in actigraphy group).

g Missing for 69 (thereof 17 in actigraphy group).

h Missing for 74 (thereof 19 in actigraphy group).

i Missing for 68 (thereof 17 in actigraphy group).

j Missing for 3 (thereof 0 in actigraphy group).

k Missing for 5 (thereof 0 in actigraphy group).

l Data in Lesotho Loti (LSL) converted to USD using the average of the 2022 and 2023 exchange rates (1 USD = 17.42 LSL).

### Primary outcomes

HIVTSQc-Teen and HIVTSQc-Parent outcomes, indicating change in treatment satisfaction, showed exclusively neutral responses or preference for DTG-based ART across all items of the respective questionnaire. Of note, 88/92 (96%) adolescents and 149/151 (99%) caregivers responded with ‘much more satisfied now’ (the most satisfied response option available) to the Satisfied item (Supplement 1 Table 3; Supplement 2 Tables 1 and 2). Among six caregivers and three adolescents who reported having experienced side effects posttransition (responding ‘yes’ to the Side effects item in the HIVTSQc), all reported a to be less bothered by side effects than before (on a scale of ‘much less bothered’ to ‘much more bothered’).

The mean estimated length of the sleep period was 9.0 h (SD: 1.0) pretransition and 9.2 h (SD: 1.0) posttransition, with a mean difference of 0.2 h (95% CI: 0.0–0.4; Table 2).

**Table 2:** Primary and secondary actigraphy-based sleep outcomes.

Outcome (*n* = 69)	Pretransition [mean (SD)]	2–4 weeks posttransition [mean (SD)]	Difference, mean (95% CI)
Co-primary			
Estimated length of sleep period (h)	9.0 (1.0)	9.2 (1.0)	0.2 (0.0 to 0.4)
Secondary			
Estimated duration of sleep in sleep period (h)	7.4 (1.1)	7.4 (1.0)	0.0 (−0.2 to 0.2)
Estimated number of awakenings during sleep window	21.0 (4.5)	22.2 (4.2)	1.2 (0.2 to 2.2)
Midpoint of sleep, clock time (SD in minutes)	1 : 55 (37 min)	1 : 55 (38 min)	0.0 (−0.1 to 0.1)
Sleep efficiency (%)^a^	77.5 (8.8)	76.7 (8.4)	−0.9 (−2.6 to 0.8)
Sleep latency^a^	0.7 (0.6)	0.6 (0.6)	−0.1 (−0.2 to 0.1)

CI, confidence interval; SD, standard deviation.

a Missing for 5 participants as sleep log data on time of going to bed was incomplete.

### Secondary outcomes

#### Questionnaires

Most caregiver-reported and self-reported responses to individual items assessing treatment satisfaction in the HIVTSQs-Teen and HIVTSQs-Parent were at or close to the upper bound of possible responses at both time points, limiting the potential to measure further improvement.

A posttransition increase in treatment satisfaction at the 0.05 significance level was observed in the HIVTSQs-Teen (*N* = 92) at 4 weeks post transition indicated by a mean of 1.398 (standard error: 0.690, *z* value; 2.025, *P* value: 0.043) with a variance of 2.562 (standard error: 1.466, *z* value: 1.747, *P* value: 0.081). No change was seen for the HIVTSQs-Parent (*N* = 137) at 4 weeks posttransition (mean: −0.103, standard error: 0.643, *z* value: −0.160, *P* value: 0.873; variance: 1.246, standard error: 0.923, *z* value: 1.350, *P* value: 0.177).

While no bothersome side effects were reported at transition, six caregivers and one adolescent reported being bothered to some degree in the Side Effects item at 4 weeks (Supplement 2 Tables 3–7).

Median GSRS-PI scores, indicating gastrointestinal symptoms, corresponded to the lower bound of the questionnaire for both the caregiver-reported and self-reported versions at both time points (Table 3). No change was observed from transition to 4 weeks posttransition for the caregiver-reported (mean difference: −0.6, 95% CI: −1.3 to 0.1) or the self-reported GSRS-PI version (mean difference: 0.4; 95% CI: −1.0 to 1.8).

Finally, self-reported symptoms of depression, measured with the CES-DC, decreased slightly at 4 weeks (mean difference: −1.4, 95% CI: −2.2 to −0.5). At transition, 24 participants (11%) scored at least 15 indicative of depression, compared with 15 (7%) at 4 weeks, 7 of whom had scores at least 15 pretransition (Table 3).

**Table 3:** Secondary self-reported tolerability outcomes.

Outcome	Measure and range	At transition, median (IQR)	At 4 weeks, median (IQR)	Difference, mean (95% CI)
Depressive symptoms, self-reported (participants ≥6 years) (*n* = 221)	CES-DC; 0 to 60 (higher scores indicate more depressive symptoms)	6 [3–11]	6 [3–9]	−1.4 (−2.2 to −0.5)
Gastrointestinal symptoms, caregiver-reported (participants <12 years) (*n* = 150)	GSRS-PI; 13–78 (13 indicates no gastrointestinal symptoms and higher scores indicate more symptoms)	13 [13–15]	13 [13–13]	−0.6 (−1.3 to 0.1)
Gastrointestinal symptoms, self-reported (participants ≥12 years) (*n* = 92)	GSRS-PI; 13–78 (higher scores indicate more gastrointestinal symptoms)	13 [13–16]	13 [13–16]	0.4 (−1.0 to 1.8)

CES-DC, Center for Epidemiological Studies Depression Scale for Children; CI, confidence interval; GSRS-PI, Gastrointestinal Symptom Rating Scale modified to the characteristics of protease inhibitors; IQR, interquartile range.

### Actigraphy measurements

The duration of time interpreted as sleep within the sleep period remained unchanged (mean difference 0.0 h, 95% CI: −0.2 to 0.2), whereas the number of periods interpreted as wakefulness during the sleep period increased slightly from pretransition to 2–4 weeks posttransition (mean difference: 1.2, 95% CI: 0.2 to 2.2; Table 2). The midpoint of sleep remained constant at 1 : 55 a.m. No difference was observed for sleep efficiency (mean difference: −0.6, 95% CI: −2.3 to 1.1) and sleep latency (mean difference −0.1, 95% CI: −0.2 to 0.1; Table 2).

These findings remained consistent comparing pretransition data to 0–2 weeks posttransition data (Supplement 1 Table 4) and analyzing only weekdays Monday through Thursday (Supplement 1 Table 5).

## Discussion

This prospective cohort study investigated treatment satisfaction, gastrointestinal and depressive symptoms and sleep in children and adolescents transitioning from LPV/r-based to DTG-based ART in Lesotho.

High treatment satisfaction was observed before and after transition, with increased satisfaction after transition among adolescents. Change-specific questionnaires (HIVTSQc-Teen/Parent) indicated a clear preference for DTG, aligning with findings in adults using the HIVTSQ [47,48] and other assessment tools [49]. This may be partly related to its once-daily dosing (in contrast to the twice-daily administration of LPV/r) and availability as a fixed-dose combination at least for some age groups and weight bands [2], reflected in a decreased pill burden from a median of five to two pills per day in our study. An apparent increase in reported ‘bother’ in the HIVTSQs-Parent and HIVTSQs-Teen Side effects item was noted; however, numbers were low (>95% reported no bother due to side effects) and all Side effects responses in the HIVTSQc were neutral or favored DTG.

DTG has been described to be well tolerated in children [20–25], an observation consistent with our findings. Albeit rare, studies in adults have linked dolutegravir to neuropsychiatric symptoms and sleep disturbances [14–19,50–53], whereas no substantial increase in depressive symptoms or sleep disturbances upon transition to DTG-based ART was observed in our study. LoDoCA was the first study to use actigraphy for objective sleep assessments in people receiving DTG. Sleep characteristics were comparable to actigraphy-based studies in similar age groups elsewhere [54], supporting the reliability of our data. Consistent with previous questionnaire-based assessments in children [21], only minor, nonclinically meaningful changes in sleep parameters were observed following DTG-transition. These included a slight increase in the sleep period length and number of awakenings, which aligns with a negative point estimate for change in sleep efficiency. No change in the estimated duration of sleep within the sleep period, sleep latency or chronotype (midpoint of sleep) was observed.

In our study, depressive symptoms slightly decreased after transition to DTG. The ODYSSEY trial showed higher, though rare, occurrences of psychiatric events and an increase in reported suicidal ideation related to DTG-based ART [21]. While our results are somewhat reassuring regarding symptoms of depression, suicidal ideation was not explicitly assessed. Finally, though gastrointestinal disturbances have previously been observed with LPV/r-based ART [8], few gastrointestinal symptoms were reported in this study, and no change upon transition to DTG was observed.

The strengths of this study include its prospective design, use of standardized, linguistically and psychometrically validated questionnaires, and objective methods for sleep assessments (actigraphy). It also demonstrates the feasibility of collecting pediatric actigraphy-based data in Lesotho and similar settings for the evaluation of sleep in the context of pediatric ART. Nevertheless, this study also has limitations. The questionnaires used were primarily developed in Europe and North America and might lack contextual or cultural appropriateness. However, this was mitigated by the comprehensive translation and linguistic validation process (including in-depth pilot testing). Actigraphy-based sleep outcomes were limited to participants with sufficient data, which could lead to nonrepresentative results if better sleep led to increased actigraphy use. This effect is hopefully somewhat mitigated by actigraphy's unobtrusiveness [55]. Actigraphy measurements furthermore were limited to nighttime, precluding daytime sleep assessment. However, this was mitigated by restricting actigraphy use to participants ≥6 years of age.

Overall, our findings suggest high tolerability of and treatment satisfaction with DTG-based ART among children and adolescents transitioning from an LPV/r-based regimen. This provides further support for the continued rollout of pediatric DTG.

## Acknowledgements

The authors would like to acknowledge Malimakatso ’Moleli, Kananelo Matlole, Lefulesele Mohaese, Neuoe Maqache, Lipuo Letsie, Pulane Maile, Itumeleng Rakuoane, and Mabutsána Ramokoatsi, all at Baylor College of Medicine Children's Foundation Lesotho, for data collection; Lipontso Motaboli, Mpho Kao, and Mathebe Kopo, all at SolidarMed, for overseeing digitalization of sleep log data; Liako Mahao as the primary translator; Joëlle Albrecht, Jannick Mauron, and Pavel Lunin for technical input on the use of actigraphy devices and analysis; and Rabia Liamlahi at the University Children's Hospital Zurich for sharing her expertise in sleep assessments in children and adolescents.

We thank Professor Bradley and her team at Health Psychology Research Ltd for their support with linguistic validation work and licenses for the Sesotho HIVTSQ-Teen and HIVTSQ-Parent status and change version questionnaires. For access to the questionnaires, please contact Health Psychology Research Ltd. at www.healthpsychologyresearch.com.

We furthermore thank all staff at Baylor College of Medicine Children's Foundation Lesotho for supporting this work. Finally, we gratefully thank and acknowledge the study participants and their caregivers.

Authors’ contributions: J.B., N.D.L., and J.A.B. designed the study with key input from F.C., M.B., T.S., J.M.B., N.T., and R.H. J.A.B. was the principal investigator. A.D., T.S., and T.M. were at various times the local principal investigator. A.D., T.S., T.M., M.B., and L.T. oversaw data collection onsite. M.B. oversaw all translations as well as linguistic validation of the Parent and Teen HIVTSQc/s. N.B.M. and T.T. ensured alignment with the national HIV programme. C.J.G. conducted the psychometric validation of the HIVTSQs/c-Teen and HIVTSQs/c-Parent questionnaires. T.L. managed the data. J.B. analyzed the data, with input from T.L., J.A.B., N.D.L., R.H., and F.C. F.C. is the responsible statistician. J.A.B. and N.D.L. acquired key funding. J.B. wrote the first draft of the manuscript. All authors reviewed and approved the manuscript.

Funding: this work was supported by the Research Fund of the University of Basel (grant number 3ZX1422 to J.A.B.) and the Swiss National Science Foundation (PCEFP3_181355 to N.D.L.). Individual co-authors were furthermore supported through further grants from the Swiss National Science Foundation (PZ00P1_201690 to J.M.B. and P500PM_221966 to J.A.B.).

### Conflicts of interest

N.D.L. reports having received travel grants to attend scientific conferences from Gilead Sciences and ViiV Healthcare. In 2022 and 2023, his division at the University Hospital Basel has received honoraria from ViiV Healthcare. C.J.G. is a director at Health Psychology Research Ltd (HPR). He is also the son of the CEO and founder of HPR, Clare Bradley. HPR owns the copyright for the HIVTSQ-Teen and HIVTSQ-Parent and similar questionnaires. HPR charges licence fees to those seeking to use its questionnaires for commercial interests. All other authors declare no conflicts of interest.

## Supplementary Material

Supplemental Digital Content

## Supplementary Material

Supplemental Digital Content

## Data Availability

De-identified participant data representing the key variables used for analysis will be made publicly available via the data repository Zenodo (https://zenodo.org/) upon publication.
