# Supplementary material for: Tolerability of lopinavir versus dolutegravir in children and adolescents with HIV
Source: AIDS. 2026 Mar 10;40(5):600–10. doi: 10.1097/QAD.0000000000004432 (PMC13034765; doi:10.1097/QAD.0000000000004432)
Supplement: Supplemental Digital Content [file aids-40-600-s001.docx]

Supplement 1:

**Supplement 1 Table 1: Baseline characteristics stratified by age category.** 3TC: lamivudine; ABC: abacavir; ART: antiretroviral therapy; AZT: zidovudine; BMI: body mass index; DTG: dolutegravir; IQR: interquartile range; LPV/r: ritonavir-boosted lopinavir; TDF: tenofovir disoproxil fumarate; USD United States Dollars; WHO: World Health Organization.

|  | **Overall**  **(n = 245)** | **Age**  **4 weeks - 6 years**  **(n = 21)** | **Age**  **6 – 12 years**  **(n = 132)** | **Age**  **> 12 years**  **(n = 92)** |
| --- | --- | --- | --- | --- |
| **General characteristics** | | | | |
| Female sex, n (%) | 115 (47) | 10 (48) | 69 (52) | 36 (39) |
| Actigraphy group, n (%) | 69 (28) | 0 (0) | 41 (31) | 28 (30) |
| Weight (kg), median [IQR]^1^ | 26.6 [21.7, 37.0] | 14.4 [12.9, 16.2] | 24.2 [20.6, 26.8] | 40.8 [34.0, 48.6] |
| Height (cm), median [IQR]^1^ | 132 [119, 146] | 99 [89, 102] | 126.2 [118.1, 133.0] | 150.2 [141.7, 156.6] |
| BMI category (WHO), n (%)^1^ |  |  |  |  |
| Severe malnutrition | 7 (3) | 0 (0) | 3 (2) | 4 (4) |
| Moderate malnutrition | 21 (9) | 0 (0) | 13 (10) | 8 (9) |
| Normal | 196 (80) | 17 (81) | 105 (80) | 74 (80) |
| Overweight | 13 (5) | 2 (10) | 9 (7) | 2 (2) |
| Obese | 7 (3) | 2 (10) | 1 (1) | 4 (4) |
| **Clinical history** | | |  |  |
| Last viral load result (copies/mL), n (%)^2^ |  |  |  |  |
| <50 | 192 (86) | 0 (NA) | 118 (91) | 74 (80) |
| 50-1000 | 20 (9) | 0 (NA) | 8 (6) | 12 (13) |
| >1000 | 10 (5) | 0 (NA) | 4 (3) | 6 (7) |
| Time since HIV diagnosis (years), median [IQR]^3^ | 10.0 [7.5, 12.7] | 3.5 [2.5, 4.7] | 8.6 [7.0, 10.0] | 13.4 [12.1, 14.9] |
| Time since ART initiation (years), median [IQR]^4^ | 9.6 [7.2, 12.4] | 3.0 [2.3, 4.7] | 8.5 [6.9, 9.8] | 13.0 [11.8, 14.8] |
| WHO stage at ART initiation, n (%)^5^ |  |  |  |  |
| Stage 1 | 156 (70) | 13 (76) | 87 (73) | 56 (66) |
| Stage 2 | 9 (4) | 1 (6) | 4 (3) | 4 (5) |
| Stage 3 | 37 (17) | 2 (12) | 20 (17) | 15 (18) |
| Stage 4 | 20 (9) | 1 (6) | 9 (8) | 10 (12) |
| CD4 count at ART initiation (cells/µL), median [IQR]^6^ | 1061 [656, 1830] | 1670 [903, 1721] | 1082 [695, 1874] | 996 [599, 1739] |
| CD4 percentage at ART initiation (cells/µL), median [IQR]^7^ | 20 [13, 29] | 26 [22, 26] | 20 [14, 29] | 20 [11, 29] |
| Immunodeficiency classification at ART initiation, n (%)^8^ |  |  |  |  |
| Not significant | 32 (18) | 0 (0) | 18 (19) | 14 (18) |
| Mild | 17 (10) | 1 (20) | 9 (9) | 7 (9) |
| Advanced | 20 (11) | 1 (20) | 10 (10) | 9 (12) |
| Severe | 108 (61) | 3 (60) | 59 (61) | 46 (61) |
| **ART before transition** | | |  |  |
| Time since start of current ART regimen (years), median [IQR]^9^ | 7.6 [4.3, 9.2] | 2.7 [2.1, 3.5] | 6.9 [3.8, 8.9] | 8.2 [7.3, 11.4] |
| ART regimen line before transition (LPV/r-regimen before transition used as first- or second-line ART), n (%)^1^ |  |  |  |  |
| First-line | 193 (79) | 21 (100) | 120 (92) | 52 (57) |
| Second-line | 51 (21) | 0 (0) | 11 (8) | 40 (43) |
| ART regimen before transition, n (%)^1^ |  |  |  |  |
| ABC/3TC/LPV/r | 169 (69) | 21 (100) | 95 (73) | 53 (58) |
| AZT/3TC/LPV/r | 71 (29) | 0 (0) | 36 (27) | 36 (39) |
| TDF/3TC/LPV/r | 3 (1) | 0 (0) | 0 (0) | 3 (3) |
| ART formulation before transition, n (%) |  |  |  |  |
| Both pills and granules | 21 (9) | 18 (86) | 3 (2) | 0 (0) |
| Only pills | 224 (91) | 3 (14) | 129 (98) | 92 (100) |
| Number of ART-items before transition, median [IQR] | 5.0 [4.5, 5.0] | 10.0 [10.0, 12.5] | 5.0 [4.4, 5.0] | 5.0 [5.0, 5.0] |
| **ART after transition** | | |  |  |
| New ART regimen line, n (%)^1^ |  |  |  |  |
| First-line | 189 (77) | 21 (100) | 119 (91) | 49 (53) |
| Second-line | 54 (22) | 0 (0) | 12 (9) | 42 (46) |
| Third-line | 1 (0) | 0 (0) | 0 (0) | 1 (1) |
| New ART regimen, n (%)^1^ |  |  |  |  |
| ABC/3TC/DTG | 176 (72) | 21 (100) | 127 (97) | 28 (30) |
| TDF/3TC/DTG | 67 (27) | 0 (0) | 3 (2) | 64 (70) |
| AZT/3TC/DTG | 1 (0) | 0 (0) | 1 (1) | 0 (0) |
| Number of ART-items after transition, median [IQR] | 2.0 [1.0, 4.0] | 5.0 [4.0, 5.0] | 4.0 [2.0, 5.0] | 1.0 [1.0, 2.0] |
| **Sociodemographic characteristics** | | |  |  |
| Primary caregiver, n (%) |  |  |  |  |
| Parent(s) | 153 (62) | 14 (67) | 87 (66) | 52 (57) |
| Other | 92 (38) | 7 (33) | 45 (34) | 40 (43) |
| Living situation, n (%) |  |  |  |  |
| With primary adult caregiver(s) | 222 (91) | 17 (81) | 122 (92) | 83 (90) |
| With other adult caregiver(s) | 11 (4) | 0 (0) | 6 (5) | 5 (5) |
| With older sibling who is < 18 years | 1 (0) | 0 (0) | 1 (1) | 0 (0) |
| Boarding School or orphanage | 11 (4) | 4 (19) | 3 (2) | 4 (4) |
| Number of household members (including participant), median [IQR] | 4 [3, 5] | 4 [3, 5] | 4 [3, 5] | 4 [3, 5] |
| Goes to school (= yes), n (%)^10^ | 224 (93) | 10 (63) | 130 (98) | 84 (91) |
| Household has regular income (= yes), n (%) | 180 (73) | 7 (33) | 96 (73) | 77 (84) |
| Average monthly household income (in USD), n (%)^11^ |  |  |  |  |
| <57 | 106 (43) | 12 (57) | 60 (45) | 34 (37) |
| 57 - 287 | 116 (47) | 9 (43) | 63 (48) | 44 (48) |
| >287 | 23 (9) | 0 (0) | 9 (7) | 14 (15) |

^1^ Missing for 1 (thereof 1 in the age group 6-12 years)

^2^  Missing for 23 (thereof 21 in the age group <6 years and 2 in the age group 6-12 years)

^3^ Missing for 11 (thereof 6 in the age group <6 years and 5 in the age group 6-12 years)

^4^ Missing for 9 (thereof 2 in the age group <6 years, 6 in the age group 6-12 years and 1 in the age group >12 years)

^5^ Missing for 23 (thereof 4 in the age group <6 years, 12 in the age group 6-12 years and 7 in the age group >12 years)

^6^ Missing for 69 (thereof 16 in the age group <6 years, 37 in the age group 6-12 years and 16 in the age group >12 years)

^7^ Missing for 74 (thereof 16 in the age group <6 years, 38 in the age group 6-12 years and 20 in the age group >12 years)

^8^ Missing for 68 (thereof 16 in the age group <6 years, 36 in the age group 6-12 years and 16 in the age group >12 years)

^9^ Missing for 3 (thereof 1 in the age group <6 years, 1 in the age group 6-12 years and 1 in the age group >12 years)

^10^ Missing for 5 (thereof 5 in the age group <6 years)

^11^ Numbers calculated based on data in Lesotho Lothi (LSL) converted to USD using the average of the 2022 and 2023 exchange rates

**Supplement 1 Table 2: Additional characteristics of actigraphy participants / measurements.** IQR: interquartile range; SD: standard deviation.

|  | **Pre-transition** | **2-4 weeks post-transition** |
| --- | --- | --- |
| **Characteristics of sleep situation** | | |
| Total number of people the participant shares a bed with, median [IQR] | 1 [0, 1] | 1 [0, 1] |
| Number of adults the participant shares a bed with, median [IQR] | 0 [0, 1] | 0 [0, 1] |
| Number of children the participant shares a bed with, median [IQR] | 0 [0, 1] | 0 [0, 1] |
| Total number of people sleeping in the same room, median [IQR] | 3 [2, 4] | 3 [2, 4] |
| **Time to bed, sleep onset and wakeup times** | | |
| Time to bed on weekdays, clock time (SD in minutes) | 20:46 (56 min) | 20:45 (47 min) |
| Sleep onset on weekdays, clock time (SD in minutes) | 21:25 (45 min) | 21:19 (49 min) |
| Wakeup on weekdays, clock time (SD in minutes) | 6:17 (55 min) | 6:26 (50 min) |
| Time to bed on weekend days, clock time (SD in minutes) | 20:53 (57 min) | 20:52 (57 min) |
| Sleep onset on weekend days, clock time (SD in minutes) | 21:27 (60 min) | 21:25 (68 min) |
| Wakeup on weekend days, clock time (SD in minutes) | 6:40 (53 min) | 6:41 (67 min) |

**Supplement 1 Table 3: Co-primary endpoint on change in treatment satisfaction.** Possible answer options range from -3 to 3 for the HIVTSQc-Parent, and from -2 to 2 for the HIVTSQc-Teen. Items are described in S2T1 and S2T2. Results are indicated as frequency (%).

|  | **Response category** | | | | | | |
| --- | --- | --- | --- | --- | --- | --- | --- |
| **Item** | **-3** | **-2** | **-1** | **0** | **1** | **2** | **3** |
| **HIVTSQc-Parent (N=151)** | | | | | | | |
| Satisfied^1^ | 0 (0%) | 0 (0%) | 0 (0%) | 0 (0%) | 0 (0%) | 2 (1%) | 149 (99%) |
| Working well^2^ | 0 (0%) | 0 (0%) | 0 (0%) | 0 (0%) | 1 (1%) | 3 (2%) | 147 (97%) |
| Own life^1^ | 0 (0%) | 0 (0%) | 0 (0%) | 0 (0%) | 0 (0%) | 2 (1%) | 149 (99%) |
| Continue^1^ | 0 (0%) | 0 (0%) | 0 (0%) | 0 (0%) | 0 (0%) | 1(1%) | 150 (99%) |
| **HIVTSQc-Teen (N=92)** | | | | | | | |
| Satisfied^1^ | NA | 0 (0%) | 0 (0%) | 2 (2%) | 2 (2%) | 88 (96%) | NA |
| Easy-Difficult^3^ | NA | 0 (0%) | 0 (0%) | 3 (3%) | 4 (4%) | 85 (92%) | NA |
| Fits your life?^1^ | NA | 0 (0%) | 0 (0%) | 2 (2%) | 5 (5%) | 85 (92%) | NA |
| Continue?^1^ | NA | 0 (0%) | 0 (0%) | 0 (0%) | 1 (1%) | 91 (99%) | NA |

^1^ Answer options: “much less satisfied now” (HIVTSQc-Parent: -3; HIVTSQc-Teen:

-2) to “much more satisfied now” (HIVTSQc-Parent: 3; HIVTSQc-Teen: 2)

^2^ Answer options: “much worse now” (-3) to “much better now” (3)

^3^ Answer options: “very more difficult now” (-2) to “much easier now” (2)

**Supplement 1 Table 4:** Actigraphy analysis comparing pre-transition to 0-2 weeks post transition data. CI: confidence interval; SD: standard deviation.

| **Outcome (n=75)** | **Pre-transition, mean (SD)** | **0-2 weeks post-transition, mean (SD)** | **Difference, mean (95% CI)** |
| --- | --- | --- | --- |
| **Co-primary** | | | |
| Estimated length of sleep period, in hours | 9.0 (1.0) | 9.1 (1.0) | 0.1 (-0.03 – 0.3) |
| **Secondary** | | | |
| Estimated duration of sleep in sleep period, in hours | 7.4 (1.2) | 7.5 (1.0) | 0.1 (-0.2 – 0.2) |
| Estimated number of awakenings during sleep window | 20.7 (4.1) | 21.8 (3.4) | 1.1 (0.4 – 1.8) |
| Midpoint of sleep, clock time (SD in minutes) | 1:50 (35 min) | 1:52 (38 min) | 0.0 (-0.1 – 0.1) |
| Sleep efficiency, in %1 | 76.9 (9.4) | 77.2 (8.5) | 0.3 (-1.9 – 2.4) |
| Sleep latency, in hours1 | 0.7 (0.6) | 0.7 (0.9) | 0.0 (-0.2 – 0.3) |

^1^ missing data for 5 participants as sleep log data on time of going to bed was incomplete

**Supplement 1 Table 5:** Actigraphy analysis of weekdays Monday to Thursday. CI: confidence interval; SD: standard deviation.

| **Outcome (n=76)** | **Pre-transition, mean (SD)** | **2-4 weeks post-transition, mean (SD)** | **Difference, mean (95% CI)** |
| --- | --- | --- | --- |
| **Co-primary** | | | |
| Estimated length of sleep period, in hours | 8.7 (1.3) | 9.0 (1.2) | 0.3 (0.1 – 0.6) |
| **Secondary** | | | |
| Estimated duration of sleep in sleep period, in hours | 7.1 (1.3) | 7.3 (1.3) | 0.2 (-0.1 – 0.4) |
| Estimated number of awakenings during sleep window | 20.3 (5.0) | 22.0 (4.5) | 1.8 (0.7 – 2.8) |
| Midpoint of sleep, clock time (SD in minutes) | 1:44 (39 min) | 1:43 (42) | 0.0 (-0.2 – 0.1) |
| Sleep efficiency, in %^1^ | 76.5 (9.3) | 77.0 (9.0) | 0.4 (-1.5 – 2.3) |
| Sleep latency, in hours^1^ | 0.8 (0.9) | 0.6 (0.7) | -0.2 (-0.4 – 0.04) |

^1^ missing data for 5 participants as sleep log data on time of going to bed was incomplete

**Supplement 1 Figure 1: Study procedures.** White background: taking LPV/r-based ART. Green background: taking DTG-based ART. DTG: dolutegravir; LPV/r: ritonavir-boosted lopinavir; VL: viral load; w: weeks.

**
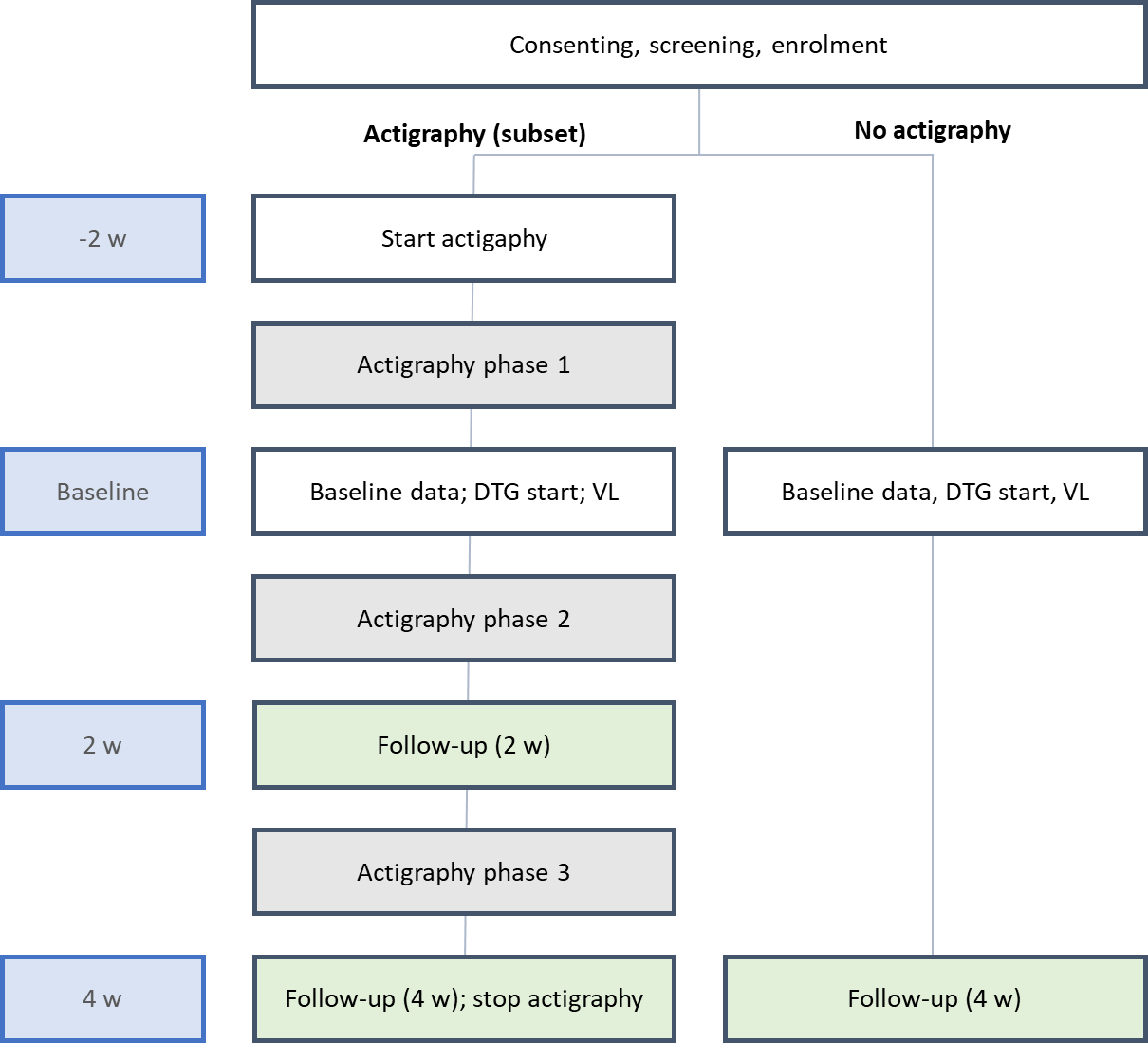
**

**Supplement 1 Figure 2: Example actigraphy plot illustrating 24-hour patterns of movement and sleep-wake behavior across 3 consecutive days.** Each panel represents one day of continuous wrist-worn accelerometer data. Colored segments indicate classified activity states. The top trace in each panel shows the angle of sensor’s z-axis relative to horizonal plane (light grey: sleep within SPT Window, turquoise: wake within SPT window, white: wakefulness). The bottom trace in each panel depicts arm movement (white: SPT Window, blue: inactivity, yellow: light PA, orange: moderate PA, red: vigorous PA, green: non-wear). SPT: sleep period time, PA: physical activity.


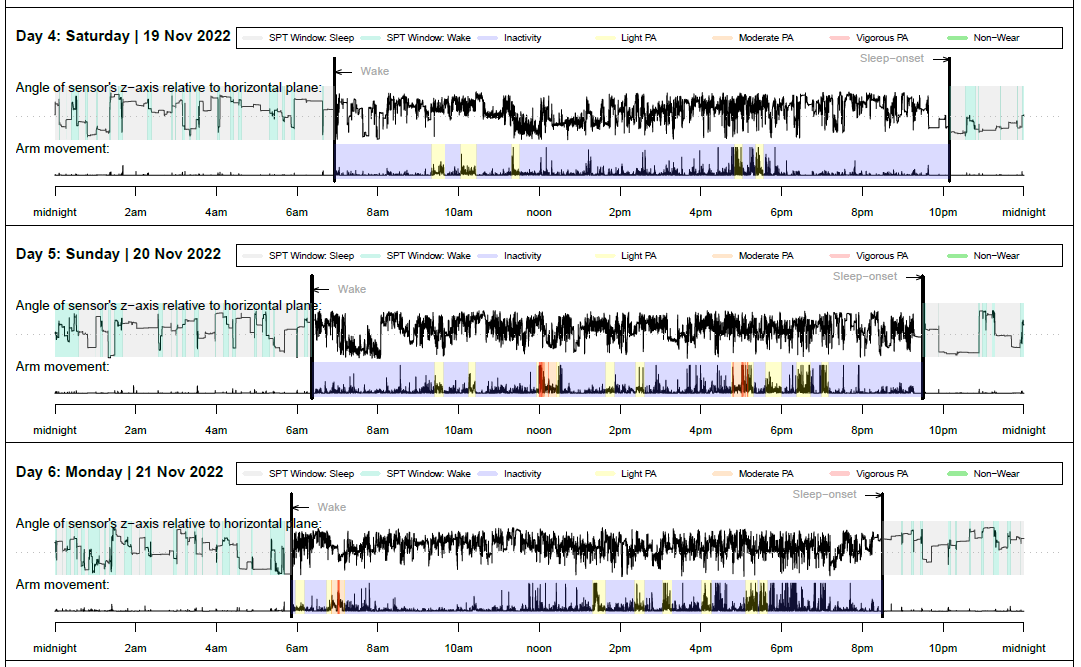


Linguistic validation

Following a standardized linguistic validation process (details of which can be found here: <https://healthpsychologyresearch.com/linguistic-validation/>), all questionnaires were independently translated from English to Sesotho by two translators (native Sesotho speakers with fluent English, or bilingual) producing two forward translations. The two forward translations were reconciled by the translation coordinator. Then, two different translators (native English speakers with fluent Sesotho, or bilingual), unaware of the original English text, backtranslated the reconciled Sesotho version into English. The two backtranslations were reviewed the coordinator and in the case of the HIVTSQs/c-Parent and -Teen questionnaires also by Health Psychology Research (HPR). Any discrepancies were discussed, and items were retranslated where necessary. The questionnaires were then reviewed by a clinician and a psychologist.

For the HIVTSQ-Parent and -Teen (status and change versions), this process was guided by HPR, including provision of one backtranslator (a first language English speaker). Items were updated based on the Clinician Report, whereupon the reviewing psychologist suggested no further changes. The HIVTSQ-Parent and -Teen (status and change versions) questionnaires were then each cognitively debriefed with 5 teens with HIV or with 5 parents of children with HIV. The Cognitive Debriefing Report was reviewed by HPR and any discrepancies were discussed with the coordinator. Where necessary items were retranslated and retested to finalize the Sesotho translation. Finally, the questionnaires were proofread.

For this project, the following trial-specific instructions were prepared which also underwent the complete linguistic validation process:

**HIVTSQc-Teen:** *For the past few weeks you have been taking medication containing dolutegravir as part of your HIV treatment.*

*We would like you to tell us how you feel about your current treatment with dolutegravir. The following questions ask you to compare it with your previous treatment with lopinavir / ritonavir medication.*

**HIVTSQc-Parent:** *For the past few weeks your child has been taking medication containing dolutegravir as part of their HIV treatment.*

*We would like you to tell us how you feel about your child’s current treatment with dolutegravir. The following questions ask you to compare it with their previous treatment with lopinavir / ritonavir medication.*
