# Supplementary material for: Tolerability of lopinavir versus dolutegravir in children and adolescents with HIV
Source: AIDS. 2026 Mar 10;40(5):600–10. doi: 10.1097/QAD.0000000000004432 (PMC13034765; doi:10.1097/QAD.0000000000004432)
Supplement: Supplemental Digital Content [file aids-40-600-s002.docx]

Supplement 2:

Psychometric validation of the HIV Treatment Satisfaction status and change questionnaire versions for caregivers and adolescents

Psychometric validation methods

Scoring of individual items

An overview of the different items of the HIVTSQs/c-Parent and HIVTSQs/c-Teen can be found in Table S2T1 and S2T2, respectively. Each item of the status version of the HIVTSQ (HIVTSQs) is scored from ‘0’ to ‘6’ for the parent version (HIVTSQs-Parent) and ‘0’ to ‘4’ for the teen version (HIVTSQs-Teen) of the questionnaire. The items Side effects and Discomfort/Pain first ask the participant if the item is relevant (e.g., ‘In recent weeks, has your child experienced any side effects of the treatment?’). If the participant responds ‘no’ then they are prompted to skip the rating scale and move on to the next item. For scoring purposes, a ‘no’ response for these items is treated as a ‘6’ on the HIVTSQs-Parent and as a ‘4’ on the HIVTSQs-Teen.

Psychometric analysis

This was the initial psychometric assessment of the HIVTSQs-Teen and -Parent. An important goal for this analysis was to determine which items are valid indicators of treatment satisfaction.

Both the HIVTSQs-Parent and -Teen were designed to measure treatment satisfaction as a single factor (or latent variable). No attempt has been made to design indicators for the questionnaires that measure related latent variables (e.g., satisfaction with clinical care). As such, this analysis was focused on identifying which items are valid indicators of treatment satisfaction in each questionnaire and then testing if there are any changes in the latent variable mean across time using the measurement invariance analysis framework [1–3].

In a first step data availability was described and distribution of the answers of individual items was assessed using frequency tables. The extent of response option usage needs to be consistent across time points for each item in ordinal factor analysis, which was assured in the current case by collapsing response categories where necessary [4]; see supplementary tables S2T3 and S2T4 to understand the extent of the collapse for caregivers. In a second step, an initial repeated-measures latent variable model was proposed for both the HIVTSQs-Parent and HIVTSQs-Teen using all items in each questionnaire. The proposed model was then assessed as to how closely it fit the data using the likelihood ratio test (or chi-square test) [5,6]. ‘Adequate fit’ was defined as a non-significant chi-square test statistic. Models were respecified as appropriate, being guided by modification indices [7] and conceptual considerations [8].

Once an adequately fitting model without parameter constraints (so-called “configural model” [6,9] ) was found, nested models were specified with progressively more stringent parameter constraints. The Wu & Estabrook [2] measurement invariance framework for categorical data was followed where threshold parameters were first constrained to be equal across the two timepoints, then loading parameters and then item intercept parameters. If the model-data fit remained adequate with all parameters constrained to equality across time, the latent variable mean differences were estimated by fixing the latent intercept at transition to 0 and the variance to 1 [5].

The R_kF_ coefficient using the multilevel.reliability function form the psych package in R [10,11] was calculated as part of the reliability analysis. The R_kF_ is one of 6 reliability coefficients presented by Shrout et al (2012) [12] and is defined as the reliability of the average ratings across all items and times (with fixed time effects).

The internal consistency was determined by calculating the omega coefficient of internal consistency for ordinal data [13] for each timepoint. Internal consistency is variously defined as how ‘homogeneous’ the items are or their ‘inter-relatedness’ of the items. Calculation of this coefficient was chosen to obtain a single statistic giving some information about how well the items are measuring a single construct.

The psychometric analysis was carried out in R version 4.4.1 using RStudio. The packages used for the analysis were lavaan (version 0.6-19 [14]), psych (version 2.4.6.26 [15]) and semTools (version 0.5-6 [16]).

Psychometric validation results

Data availability

For the caregivers answering the HIVTSQs-Parent, there were 4/147 missing data points accounted for by 3 participants. These were instances where responses indicated that he or she preferred not to respond (PNTR) to the item. Two caregivers responded PNTR to the Working well item, one only at transition and the other only at 4 weeks post-transition. One other caregiver responded PNTR to the Family life item and Own life item. A further 7 of the remaining 144 caregivers needed to be excluded. Due to a technical error, these caregivers were presented, at transition, with a draft version of the questionnaire being adapted for electronic use. In total, 137 caregivers who completed the HIVTSQs-Parent could be included in the latent variable modelling and reliability analyses.

For the HIVTSQs-Teen, 1 PNTR response was given to the Side effects item at transition and 2 PNTR responses were given to the Discomfort/Pain item at four weeks post-transition. As these items were excluded from the initial specification for the latent variable model of the HIVTSQs-Teen due to lack of variance (see next paragraph), missing data did not affect the sample size for the latent variable analysis of the HIVTSQs-Teen data.

Data distribution

Data from both the HIVTSQs-Parent was highly skewed. Asymmetric responses to the status version are expected [17], but the asymmetry was perhaps more pronounced than has been observed with other samples with treatment satisfaction questionnaires for other conditions. The asymmetry was so high in this case that the response categories needed to be collapsed down, for half the items, to just three: 6 (the most satisfied response option), 5 and less than 5. The same was done for the HIVTSQs-Teen data, for which the scale range of each item was mostly limited to three categories. Uncollapsed data of the HIVTSQs-Parent at transition is shown in Table S2T3. Collapsed data of both the HIVTSQs-Parent and HIVTSQs-Teen for both at transition and 4 weeks post transition is shown in tables S2T4-S2T7).

Latent variable modelling

All items were initially specified as reflective indicators of treatment satisfaction except the items Side effects and Discomfort/Pain. For both the HIVTSQs-Parent and -Teen, these items showed a complete or near-complete lack of variance at least at one timepoint (see Table S2T3-S2T7).

For both the HIVTSQs-Parent and -Teen, the initial specification produced either an inadmissible solution (e.g., extremely high scaling factor, negative item variance) or one where the model-data fit was inadequate. There was more than one re-specification step required for both the HIVTSQs-Parent and -Teen analysis before adequate model-data fit was reached.

HIVTSQs-Parent

The initial latent variable model of the HIVTSQs-Parent included items: Satisfied, Working well, Easy-Difficult, Fits with life, Family life, Own life, Understanding, and Continue. The final re-specification used four items: Satisfied, Working well, Own life, and Continue.

The items Understanding, Easy-Difficult, Fits with life and Family life, were excluded from the latent variable model for the HIVTSQs-Parent for the following reasons.

The Understanding item produced high modification indices (MIs) [18] with the responses at the second timepoint associating strongly with responses to all items at transition (MI = 308) and vice versa (MI = 115). These associations are difficult to explain in real-world terms because the Understanding item is not like an overview item (asking, for example, how satisfied are you with your current treatment?). Understanding of disease or treatment is not likely to be indicative of treatment satisfaction in the same manner as an item asking about, for example, perceived treatment efficacy. As perceived treatment efficacy changes, treatment satisfaction is only likely to change in the same way. The association with Understanding and treatment satisfaction is less clearcut. Understanding may be high or increase, but it does not follow that, in every case, treatment satisfaction will then also be high or will increase.

The Easy-Difficult item also contributed substantially to model-data misfit, having a high MI with other items across time (MI = 212). This item has been designed to capture concepts such as convenience and flexibility, but it may also be seen in some contexts as being little different from the Satisfied item. Unless correlated errors are specified between two overlapping items, model-data fit will remain inadequate. However, there is often little to be gained from specifying correlated errors between two overlapping items. It is better to aim for model simplification and reduce the number of unnecessary items.

The Fits with life item associated with other items across time and contributed to poor model-data fit (MI = 226). This item was derived from the Fits with lifestyle item for the adult HIVTSQ. The Fits with lifestyle item may work well for adults in some contexts, but its equivalent about fitting in with the child’s life, in the context of the HIVTSQs-Parent, may seem like another generic question like the Satisfaction or Easy-Difficult item.

The Family life item associated strongly with the Own life item. For many caregivers, there will understandably be a strong overlap between family life and their own life, while for others there may be important distinctions. Allowing for correlated errors between these items would seem like a reasonable re-specification, but instead the final re-specification excludes the Family life item and retains the Own life item.

HIVTSQs-Teen

The initial specification for the latent variable model of the of the HIVTSQs-Teen used items: Satisfied, Working well, Easy-Difficult, Fits your life, Understanding, and Continue. The final specification for the configural model for the adolescents used items: Satisfied, Easy-Difficult, Fits your life and Continue. The items Working well and Understanding were excluded from the latent variable model for the HIVTSQs-Teen for the following reasons.

The Working well item produced an inadmissible solution when included, with a negative variance of -0.194 at the second timepoint. This item covers perception of efficacy which is generally considered to be an important aspect of treatment satisfaction. However, in some cases respondents may not feel confident in their perception. This may have been the case here where only four weeks elapsed before the adolescents were asked to report on perceived efficacy of the treatment.

Two associations with high MIs involved the Understanding item. Also, as stated above, it makes little conceptual sense to specify this item as an indicator. An Understanding item would be better placed as a formative indicator of treatment satisfaction or a reflective indicator of another latent variable interacting with treatment satisfaction in a more complex model with a structural component.

Measurement invariance testing

Measurement invariance testing following the Wu & Estabrook framework confirmed that for both (caregiver and adolescent) models constraining threshold, loading and item intercept parameters across time points did not significantly affect model-data fit. Therefore, both models were used to estimate latent variable mean differences and variances as described in the main text, with the transition time point set as the reference where latent means and variances are fixed at 0 and 1, respectively.

Reliability

The R_kF_ = 0.57 for caregivers and R_kF_ = 0.64 for adolescents. According to the authors of this reliability coefficient [19] these values represent ‘fair’ and ‘moderate’ levels of reliability, respectively.

Internal consistency

The omega coefficient for categorical data (Ω_cat_) [13] for the HIVTSQs-Parent was 0.623 [95% CI: 0.316 - 0.736] at transition and 0.812 [95% CI: 0.673-0.896] at 4 weeks post-transition. For the HIVTSQs-Teen the coefficients were 0.673 [95% CI: 0.420-0.804] and 0.722 [95% CI: 0.432-0.851] for transition and 4 weeks post-transition respectively. The commonly accepted heuristic for ‘acceptable’ internal consistency is 0.7. The Ω_cat_ is <0.7 at transition for both caregivers and adolescents, but is >0.7 in both cases at 4 weeks post transition. As mentioned, Tables S2T3-S2T7 indicate that the data is highly skewed. With such highly asymmetric response patterns, the true score variance is very low and only a very small level of error variance will be needed to suppress the internal consistency coefficient.

Psychometric validation discussion

This study reports the first use of the HIVTSQ-Parent and -Teen with both the status and change versions being used. Latent variable modelling [6] was applied to the data with measurement invariance analysis used to test the consistency of the items as indicators of treatment satisfaction over a period of four weeks.

For both the HIVTSQs-Parent and -Teen, four items were identified as robust indicators of treatment satisfaction. For the HIVTSQs-Parent these items were: Satisfied, Working well, Own life and Continue. For the HIVTSQs-Teen, the 4 indicators of treatment satisfaction are: Satisfied, Easy-Difficult, Fits your life, and Continue. Simultaneously, measurement invariance analysis could use these four items to assess changes in treatment satisfaction as a latent variable.

Even though the Side effects and Discomfort/Pain items could not be included in the latent variable model given the lack of variance in the responses at one timepoint, it is still important to consider the Side effects and Discomfort/pain items as standalone items. These are potentially important aspects of treatment satisfaction, but in the current sample, more than 95% of participants did not recognize any side effects or discomfort/pain at either of the recorded timepoints. Considering the frequency tables, it is clear that 6 of 147 caregivers reported increased bother from side effects at 4 weeks post-transition. For 3 of these participants, the bother was substantial. They reported being ‘very bothered’ by side effects or close to that. Interestingly, similar numbers of adolescents reported ‘not at all bothered’ at transition and at four weeks post-transition. 91 of 92 adolescents reported no side effects or ‘not at all bothered’ by side effects at transition and 4 weeks post-transition.

Researchers can use the 4 indicators identified here as indicators of treatment satisfaction for caregivers (items Satisfied, Working well, Own life and Continue) and adolescents (items Satisfied, Easy-Difficult, Fits your life and Continue). Many will be familiar with sum scoring methods. This is an intuitive and accessible approach that is still seen as useful [20]. However, researchers should be aware that assuming metric properties for ordinal scales (such as those used in the HIVTSQs-Parent and -Teen) has considerable pitfalls [21]. Multilevel ordinal regression is regarded by some as a more robust approach than sum scoring that avoids the metric assumption [22]. Both sum scores and multilevel ordinal regression avoid the need to carry out latent variable modelling.

It remains to be seen whether these items identified here are robust indicators for other samples. More latent variable modelling work is needed to test if the items identified here as valid indicators of treatment satisfaction generalize to other samples. It would be a valuable exercise in future work to apply similar analysis methods to an independent sample and observe if the same or similar re-specifications are warranted. This will inform understanding of how generalizable the re-specifications made here are to the wider patient and caregiver population.

Psychometric validation limitations

*A priori* sample size planning was not carried out for psychometric validation. Didactic papers describing such approaches have been available for some time (e.g. [23]) and are becoming increasingly accessible [24]. Although such methods are still challenging to implement particularly if it is accepted that non-normal data (such as that produced by the ordinal scales of the HIVTSQs-Parent and -Teen) need genuinely non-normal data to be simulated to support such planning [25].

This is a single sample where participants did not complete the questionnaires unaided. An interviewer assisted participants in completing the -Parent and -Teen questionnaires. The questionnaires are designed for the participant to complete by themselves. Ideally, the participant should understand that their responses will be anonymous. If participants believe their responses might negatively impact their care, then this is likely to exaggerate the ceiling effects known to characterize the status version [17].

References

1 Meredith W. **Measurement invariance, factor analysis and factorial invariance**. *Psychometrika* 1993; **58**:525–543.

2 Wu H, Estabrook R. **Identification of Confirmatory Factor Analysis Models of Different Levels of Invariance for Ordered Categorical Outcomes**. *Psychometrika* 2016; **81**:1014–1045.

3 Svetina D, Rutkowski L, Rutkowski D. **Multiple-Group Invariance with Categorical Outcomes Using Updated Guidelines: An Illustration Using Mplus and the lavaan/semTools Packages**. *Struct Equ Modeling* 2020; **27**:111–130.

4 DiStefano C, Shi D, Morgan GB. **Collapsing Categories is Often More Advantageous than Modeling Sparse Data: Investigations in the CFA Framework**. *Struct Equ Modeling* 2021; **28**:237–249.

5 Rick H. Hoyle. *Handbook of Structural Equation Modeling Second Edition*. Second Edition. Guildford Press; 2023.

6 Kenneth A. Bollen. *Structural Equations with Latent Variables*. John Wiley & Sons, Inc.; 1989.

7 Sörbom D. **Model modification**. *Psychometrika* 1989; **54**:371–384.

8 Rhemtulla M, van Bork R, Borsboom D. **Worse than measurement error: Consequences of inappropriate latent variable measurement models.** *Psychol Methods* 2020; **25**:30–45.

9 Somaraju A V., Nye CD, Olenick J. **A Review of Measurement Equivalence in Organizational Research: What’s Old, What’s New, What’s Next?** *Organ Res Methods* 2022; **25**:741–785.

10 Revelle W, Wilt J. **Analyzing dynamic data: A tutorial**. *Pers Individ Dif* 2019; **136**:38–51.

11 Revelle W, Condon DM. **Reliability from α to ω: A tutorial**. *Psychol Assess* 2019; **31**:1395–1411.

12 Mehl MR, & CTS. *Handbook of research methods for studying daily life*. The Guilford Press.; 2012.

13 Green SB, Yang Y. **Reliability of summed item scores using structural equation modeling: An alternative to coefficient alpha**. *Psychometrika* 2009; **74**:155–167.

14 Rosseel Y. **lavaan: An R Package for Structural Equation Modeling**. *J Stat Softw* 2012; **48**:1–36.

15 **Package “psych” Title Procedures for Psychological, Psychometric, and Personality Research**. Published Online First: 2025.https://personality-project.org/r/psych/ (accessed 7 Apr2025).

16 Jorgensen TD, Pornprasertmanit S, Schoemann AM, Rosseel Y. **semTools: Useful Tools for Structural Equation Modeling**. *CRAN: Contributed Packages* Published Online First: 18 May 2012. doi:10.32614/CRAN.PACKAGE.SEMTOOLS

17 Bradley C. **Diabetes treatment satisfaction questionnaire. Change version for use alongside status version provides appropriate solution where ceiling effects occur**. *Diabetes Care* 1999; **22**:530–532.

18 Sörbom D. **Model modification**. *Psychometrika 1989 54:3* 1989; **54**:371–384.

19 Shrout PE, Lane SP. **Reliability.** *APA handbook of research methods in psychology, Vol 1: Foundations, planning, measures, and psychometrics* 2012; :643–660.

20 Widaman KF, Revelle W. **Thinking thrice about sum scores, and then some more about measurement and analysis**. *Behav Res Methods* 2023; **55**:788–806.

21 Liddell TM, Kruschke JK. **Analyzing ordinal data with metric models: What could possibly go wrong?** *J Exp Soc Psychol* 2018; **79**:328–348.

22 Bürkner PC, Vuorre M. **Ordinal Regression Models in Psychology: A Tutorial**. *Adv Methods Pract Psychol Sci* 2019; **2**:77–101.

23 Muthén LK, Muthén BO. **How to Use a Monte Carlo Study to Decide on Sample Size and Determine Power**. *Structural Equation Modeling* 2002; **9**:599–620.

24 Goretzko D, Siemund K, Sterner P. **Evaluating Model Fit of Measurement Models in Confirmatory Factor Analysis**. *Educ Psychol Meas* 2024; **84**:123–144.

25 Grønneberg S, Foldnes N. **Covariance Model Simulation Using Regular Vines**. *Psychometrika* 2017; **82**:1035–1051.

Tables

Table S2T1: Overview table showing HIVTSQs/c-Parent items

| Item Number | Item Shortname | Description* |
| --- | --- | --- |
| 1 | Satisfied | Level of general satisfaction with the child’s current treatment. |
| 2 | Working well | Asking about perceived efficacy of child’s treatment. |
| 3 | Side effects | Asking about bother caused by side effects (if applicable) |
| 4 | Easy-Difficult | Asking how easy or difficult the treatment is. |
| 5 | Fits with life | Level of satisfaction with how well the treatment fits in with the child’s life. |
| 6 | Family life | Level of satisfaction with how the treatment affects family life. |
| 7 | Own life | Level of satisfaction with how treatment affects the caregiver’s life. |
| 8 | Understanding | Level of satisfaction with understanding of child’s HIV. |
| 9 | Discomfort/Pain | Asking about bother caused by discomfort or pain (if applicable). |
| 10 | Continue | Level of satisfaction at the prospect of the child continuing with current treatment. |

* Questionnaire items have not been presented verbatim. This is to protect against misuse of the questionnaires and proliferation of unauthorized versions. Please contact Health Psychology Research Ltd for more information on the HIVTSQ-Teen and -Parent.

Table S2T2: Overview table showing HIVTSQs/c-Teen items

| Item Number | Item Shortname | **Description*** |
| --- | --- | --- |
| 1 | Satisfied | Level of general satisfaction with the participant’s current treatment. |
| 2 | Working well | Asking about perceived efficacy of child’s treatment. |
| 3 | Side effects | Asking about bother caused by side effects (if applicable) |
| 4 | Easy-Difficult | Asking how easy or difficult the treatment is. |
| 5 | Fits your life | Level of satisfaction with how well the treatment fits in with the participant’s life. |
| 6 | Understanding | Level of satisfaction with understanding of participant’s HIV. |
| 7 | Discomfort/Pain | Asking about bother caused by discomfort or pain (if applicable). |
| 8 | Continue | Level of satisfaction at the prospect of the participant continuing with current treatment. |

* Questionnaire items have not been presented verbatim. This is to protect against misuse of the questionnaires and proliferation of unauthorized versions. Please contact Health Psychology Research Ltd for more information on the HIVTSQ-Teen and -Parent.

Table S2T3: Frequency table showing HIVTSQs-Parent responses at transition (no collapse)

| **Items** | **Response categories** | | | | | | | |
| --- | --- | --- | --- | --- | --- | --- | --- | --- |
|  | **0** | **1** | **2** | **3** | **4** | **5** | **6** |  |
| Satisfied | 1 | - | - | - | - | 10 | 136 |  |
| Working well | 1 | - | 1 | - | 1 | 9 | 134 |  |
| Side effects | - | - | - | - | - | - | 147 |  |
| Easy-Difficult | 1 | 1 | - | 3 | 15 | 19 | 108 |  |
| Fits with life | - | - | - | - | 1 | 11 | 135 |  |
| Family life | - | - | - | - | 2 | 8 | 136 |  |
| Own life | - | - | - | 1 | 2 | 9 | 134 |  |
| Understanding | - | - | - | - | 2 | 9 | 136 |  |
| Discomfort/Pain | - | - | - | - | - | - | 147 |  |
| Continue | 1 | - | - | 1 | 1 | 9 | 135 |  |

Table S2T4: Frequency table showing HIVTSQs-Parent responses at transition (collapsed categories)

| **Items** | **Response categories** | | | | | | | |
| --- | --- | --- | --- | --- | --- | --- | --- | --- |
|  | **0** | **1** | **2** | **3** | **4** | **5** | **6** |  |
| Satisfied | - | - | - | - | 1 | 10 | 136 |  |
| Working well | - | - | - | 2 | 1 | 9 | 134 |  |
| Side effects | - | - | - | - | - | - | 147 |  |
| Easy-Difficult | - | - | 2 | 3 | 15 | 19 | 108 |  |
| Fits with life | - | - | - | - | - | 12 | 135 |  |
| Family life | - | - | - | - | 2 | 8 | 136 |  |
| Own life | - | - | - | - | 3 | 9 | 134 |  |
| Understanding | - | - | - | - | 2 | 9 | 136 |  |
| Discomfort/Pain | - | - | - | - | - | - | 147 |  |
| Continue | - | - | - | - | 3 | 9 | 135 |  |

Table S3T5: Frequency table showing HIVTSQs-Parent responses at 4 weeks post-transition (collapsed categories)

| **Items** | **Response categories** | | | | | | | |
| --- | --- | --- | --- | --- | --- | --- | --- | --- |
|  | **0** | **1** | **2** | **3** | **4** | **5** | **6** |  |
| Satisfied | - | - | - | - | 4 | 11 | 132 |  |
| Working well | - | - | - | 2 | 3 | 11 | 130 |  |
| Side effects* | 2 | 1 | - | 2 | - | 1 | 141 |  |
| Easy-Difficult | - | - | 3 | 2 | 4 | 10 | 128 |  |
| Fits with life | - | - | - | - | - | 5 | 142 |  |
| Family life | - | - | - | - | 1 | 5 | 141 |  |
| Own life | - | - | - | - | 1 | 5 | 141 |  |
| Understanding | - | - | - | - | 1 | 5 | 141 |  |
| Discomfort/Pain* | - | 1 | - | - | - | - | 146 |  |
| Continue | - | - | - | - | 4 | 8 | 135 |  |

*Side-effects and Discomfort/Pain were not collapsed.

Table S2T6: Frequency table showing HIVTSQs-Teen responses at transition (collapsed categories)

| **Items** | **Response categories** | | | | |
| --- | --- | --- | --- | --- | --- |
|  | **0** | **1** | **2** | **3** | **4** |
| Satisfaction | - | - | 3 | 11 | 78 |
| Working well | - | - | 4 | 17 | 71 |
| Side effects | - | - | - | - | 91 |
| Easy-Difficult | - | 3 | 5 | 20 | 64 |
| Fits your life | - | - | 1 | 10 | 81 |
| Understanding | - | - | - | 19 | 73 |
| Discomfort/Pain | - | - | - | 3 | 89 |
| Continue | - | - | 8 | 15 | 69 |

Table S2T7: Frequency table showing HIVTSQs-Teen responses at 4 weeks post-transition (collapsed categories)

| **Items** | **Response categories** | | | | |
| --- | --- | --- | --- | --- | --- |
|  | **0** | **1** | **2** | **3** | **4** |
| Satisfaction | - | - | 3 | 10 | 79 |
| Working well | - | - | 1 | 8 | 83 |
| Side effects | - | - | 1 | - | 91 |
| Easy-Difficult | - | 4 | 2 | 7 | 79 |
| Fits your life | - | - | 2 | 7 | 83 |
| Understanding | - | - | - | 10 | 82 |
| Discomfort/Pain | - | - | - | 1 | 89 |
| Continue | - | - | 3 | 6 | 83 |
